# The Relationship between Endothelial Progenitor Cell Populations and Epicardial and Microvascular Coronary Disease—A Cellular, Angiographic and Physiologic Study

**DOI:** 10.1371/journal.pone.0093980

**Published:** 2014-04-15

**Authors:** Kim H. Chan, Philippa J. L. Simpson, Andy S. Yong, Louise L. Dunn, Chirapan Chawantanpipat, Chijen Hsu, Young Yu, Anthony C. Keech, David S. Celermajer, Martin K. C. Ng

**Affiliations:** 1 Department of Cardiology, Royal Prince Alfred Hospital, Sydney, New South Wales, Australia; 2 The Heart Research Institute, Sydney, New South Wales, Australia; 3 Sydney Medical School, The University of Sydney, Sydney, New South Wales, Australia; 4 Department of Cardiology, Concord Hospital, Sydney, New South Wales, Australia; 5 National Health and Medical Research Council Clinical Trials Centre, Sydney, New South Wales, Australia; Center for Interdisciplinary Research in Biology (CIRB) is a novel Collège de France/CNRS/INSERM, France

## Abstract

**Background:**

Endothelial progenitor cells (EPCs) are implicated in protection against vascular disease. However, studies using angiography alone have reported conflicting results when relating EPCs to epicardial coronary artery disease (CAD) severity. Moreover, the relationship between different EPC types and the coronary microcirculation is unknown. We therefore investigated the relationship between EPC populations and coronary epicardial and microvascular disease.

**Methods:**

Thirty-three patients with a spectrum of isolated left anterior descending artery disease were studied. The coronary epicardial and microcirculation were physiologically interrogated by measurement of fractional flow reserve (FFR), index of microvascular resistance (IMR) and coronary flow reserve (CFR). Two distinct EPC populations (early EPC and late outgrowth endothelial cells [OECs]) were isolated from these patients and studied *ex vivo*.

**Results:**

There was a significant inverse relationship between circulating OEC levels and epicardial CAD severity, as assessed by FFR and angiography (r = 0.371, p = 0.04; r = -0.358, p = 0.04; respectively). More severe epicardial CAD was associated with impaired OEC migration and tubulogenesis (r = 0.59, p = 0.005; r = 0.589, p = 0.004; respectively). Patients with significant epicardial CAD (FFR<0.75) had lower OEC levels and function compared to those without hemodynamically significant stenoses (p<0.05). In contrast, no such relationship was seen for early EPC number and function, nor was there a relationship between IMR and EPCs. There was a significant relationship between CFR and OEC function.

**Conclusions:**

EPC populations differ in regards to their associations with CAD severity. The number and function of OECs, but not early EPCs, correlated significantly with epicardial CAD severity. There was no relationship between EPCs and severity of coronary microvascular disease.

## Introduction

Endothelial progenitor cells (EPCs) have been implicated in protection against vascular injury and atherogenesis [1]. Animal experiments have reported that EPC transplantation prevents progression of atherosclerosis [2]. Given their potential for cardiovascular regeneration and repair, studies have also explored whether endogenous circulating EPC levels may be a marker of “vascular health”. However, studies exploring the relationship between circulating EPC levels and angiographic severity of epicardial coronary artery disease (CAD) have yielded conflicting results [3], [4]. Possible reasons for these include variable EPC definitions [5] and exclusive reliance on angiography alone to assess CAD severity, a technique which may have limited correlation with the hemodynamic significance of CAD [6]. Moreover, little is known about EPC relationships with microvascular disease, a critical determinant of cardiovascular outcomes [7].

There is currently no uniform definition of an EPC [5], [8]. It has been proposed that cells expressing surface protein markers CD34, KDR and/or CD133 may represent the putative EPC, although these markers are also present in hematopoietic progenitor cells [9]. Alternatively, EPCs are isolated by culture-based techniques and are classified according to their morphology and the time at which they appear in culture: early EPCs typically appear in culture after 4 to 7 days, and are similar to those originally described by Asahara *et al* [1]; in contrast, late outgrowth endothelial cells (OECs, also known as late EPCs or endothelial colony forming cells, ECFCs) appear after 14 to 21 days of culture [10]. Early EPCs and OECs originate from different bone marrow-derived mononuclear cell populations. Early EPCs are derived from a heterogenous population of CD45^+^ hematopoietic cells, including CD34^+^/CD45^+^ hematopoietic progenitors and CD45^+^/CD14^+^ monocytic cells [11]. In contrast, OECs are a homogenous population of rare circulating cells that derive from non-hematopoietic non-monocytic CD45^−^/CD14^−^ cells [12], [13]. In fact, recent studies reported that these culture-derived EPC populations exhibit different biological characteristics, with OECs rather than early EPCs directly incorporating into vascular networks *in vitro* [14] and forming perfused vessels *in vivo* [15].

Advances in coronary guide wire technology have facilitated the development of physiologic indices to allow accurate assessment of coronary epicardial arteries and the microcirculation [16]. The fractional flow reserve (FFR) and index of microvascular resistance (IMR) are specific measures of epicardial disease and integrity of the microcirculation respectively, whereas coronary flow reserve (CFR) provides a functional measure of both levels of the circulation [16]. Given the different biological characteristics between EPC populations, we hypothesized that distinct EPC populations may play different roles in atheroprotection, and therefore explored whether the different EPC populations would be related to key measures of coronary epicardial and microvascular disease in humans by combining cellular, angiographic and physiologic assessments.

## Methods

### Study participants

We prospectively recruited consecutive patients referred for elective coronary angiography for investigation of chest pain which was suspicious for stable angina pectoris. Only patients with a spectrum of left anterior descending artery (LAD) disease without significant disease elsewhere in the epicardial coronary circulation were included in this study. Patients were excluded if they have had an acute coronary syndrome, previous coronary artery bypass surgery, end-stage renal disease, anemia, were on dipyridamole or methylxanthine treatment, or were pregnant at time of study. Baseline clinical characteristics were obtained. This study had ethics committee approval in accordance with the Declaration of Helsinki and Good Clinical Practice Guidelines (Sydney South West Area Health Service Ethics Approval #X06-0191). All study participants provided written informed consent.

### Assessment of coronary artery disease severity

Physiologic assessment of CAD severity was performed as previously described [16]. Briefly, intracoronary nitroglycerin (200 µg) was administered prior to physiology assessment. A 0.014-in pressure-temperature sensor guide wire (Certus Pressure Wire, St. Jude Medical, Minnesota) was advanced into the distal LAD. Mean transit time (Tmn) at rest was measured by the thermodilution method. Maximal steady-state hyperemia was induced by adenosine infusion (140 µg/kg/min) and simultaneous measurements of Tmn, proximal (Pa) and distal (Pd) arterial pressure during hyperemia were obtained. Measurements of the fractional flow reserve (FFR = Pd/Pa) were obtained to assess the hemodynamic significance of epicardial CAD. The coronary flow reserve (CFR = rest Tmn/hyperemic Tmn) was measured to assess the hemodynamic significance of both epicardial and microvascular CAD. Of note, three patients had CFR<1.0, likely due to coronary steal during adenosine infusion in the context of hemodynamically critical LAD stenoses (FFR<0.5; stenosis severity ≥90%) [17]. Patients with hemodynamically significant epicardial CAD (FFR<0.75) underwent angioplasty, and the wedge pressure was recorded during first balloon inflation. The index of microvascular resistance (IMR), a measure of hemodynamic significance of microvascular CAD was calculated using the formula Pa × hyperemic Tmn × ([Pd–wedge pressure]/[Pa–wedge pressure]). In patients not undergoing angioplasty, IMR was calculated using the simplified formula Pd/hyperemic Tmn.

For angiographic assessment of epicardial CAD severity, stenosis severity was estimated by visual assessment. The modified Gensini index was also calculated for all patients as previously described [18]. Briefly, this index assigns a heavier weight depending on stenosis severity and vessel segment size and importance. The modified Gensini score is derived from the sum of the product of stenosis severity grading and vessel segment weight for each coronary segment. For angiographic assessment of the coronary microcirculation, the corrected Thrombolysis in Myocardial Infarction (TIMI) frame count and TIMI myocardial perfusion grade were assessed [19], [20]. Personnel involved in EPC isolation and functional assay assessment were blinded to the details of baseline patient variables, coronary physiological and angiographic measurements.

### EPC isolation, cultivation and quantification

EPCs were identified based on cell culture and flow cytometry methods as previously described [14]. Briefly, ∼50 mL of peripheral venous blood was obtained at the beginning of the coronary angiography procedure. Mononuclear cells were then isolated via density gradient separation using Lymphoprep (Axis-Shield, Oslo, Norway). To obtain early EPCs, 2×10^7^ mononuclear cells per well were seeded onto 6-well tissue culture plates pre-coated with human fibronectin (BD Biosciences) and cultured in endothelial cell growth medium-2 (EGM-2; Lonza Walkersville Inc., Maryland) supplemented with 10% fetal bovine serum. After 24 hours, non-adherent cells were discarded. The cells were assessed for the ability to ingest 1,1′dioctadecyl-3,3,3′,3′-tetramethyl-indocarbocyanide-labeled acetylated low-density lipoprotein (4 µg/mL, Invitrogen, California) and to bind fluorescein isothiocyanate-*Ulex europaeus* agglutinin lectin (10 µg/mL, Sigma, Missouri) after 7 days of culture. The cells were then enumerated with 10 random fields of view per well at 100× magnification.

To obtain OECs, between 1-2×10^7^ mononuclear cells were plated out as above on tissue culture plates pre-coated with type I collagen I (BD Biosciences). After 21 days of culture, the number of OEC colonies was quantified by scanning culture plates as previously described [3]. Two patients did not have sufficient mononuclear cells isolated to allow plating for OECs.

### Flow cytometry analysis

EPCs were also quantified using flow cytometry as previously described [14] to allow assessment of CD34^+^/KDR^+^ EPCs and enriched populations of early EPCs (CD34^+^/CD45^+^) and OECs (CD34^+^/CD45^−^) [9]. Briefly, cells isolated from whole blood by density gradient separation were gated by forward and side scatter to select mononuclear cells by excluding erythrocytes, granulocytes and cell debris. The mononuclear-gated cells were then incubated with the following antibodies against human antigens: fluorescein isothiocyanate-conjugated CD34 (BD Pharmingen, California), phycoerythrin-conjugated KDR (R&D Systems, Minnesota), and phycoerythrin-Cy5-conjugated CD45 (BD Pharmingen). Fluorescent isotype-matched IgG1 antibodies were used as negative controls. Each analysis was performed in duplicates and included 250,000 events. The coefficients of variation for the EPC populations studied were previously reported from our laboratory to be <15% [21]. Flow cytometry was not performed on the first four patients.

### EPC angiogenic functional assays

EPC functional assays were performed on early EPCs at 7 days after isolation and on OEC after expansion to passage 2-3.

#### Migration assay

Migration was assessed for both early EPCs and OECs using a transwell (Boyden) chamber assay (Costar, Massachusetts). Briefly, 1×10^4^ EPCs suspended in serum-free endothelial basal medium (Lonza) were placed in the transwell. The transwell was then placed in a 24-well culture dish (equipped with a 12-mm-diameter polyester membrane with pore size 8 µm) containing EGM-2 and 10% fetal bovine serum as previously described [22]. After 24 hours incubation at 37°C, the lower side of the transwell membrane was stained with *Ulex europaeus* lectin (10 µg/mL) and DAPI. Each assay included 4 replicates, and EPCs migrating into the lower chamber were counted with 5 random fields of view per well at 100× magnification. Seven patients did not have OEC migration assay performed due to insufficient cell numbers or cell culture contamination.

#### Tubulogenesis assay

Tubulogenesis was assessed for both early EPCs and OECs using Matrigel (BD Biosciences). Briefly, 4×10^3^ EPCs were seeded in each well of growth factor-reduced Matrigel-coated 96-well plate. Given that early EPCs have been shown to augment angiogenesis in a paracrine fashion, but do not form tubules independently [14], early EPCs were also co-cultured with 4×10^3^ human umbilical vein endothelial cells (HUVECs), with one analysis consisting of HUVEC alone as control. At least 6 replicates were performed for each analysis. After 5 hours incubation at 37°C, the number of connections between tubule nodes was counted. Eleven patients did not have OEC tubulogenesis assay performed due to issues outlined above.

#### Proliferation assay

OEC proliferation was assessed using the Click-iT EdU Cell Proliferation Assay (Invitrogen, Carlsbad, California) as per manufacturer's instructions. Briefly, 5×10^4^ OECs were seeded into each well of a 12-well plate. Modified nucleoside (5-ethynyl-2′-deoxyuridine; EdU) was added and the cells incubated overnight at 37°C. The incorporation of EdU into the DNA of newly proliferated cells was measured by intracellular binding of an AlexaFluor-488 probe and flow cytometric quantification.

### Growth factor assessment

The cytokines vascular endothelial growth factor (VEGF_165_) and stromal cell-derived factor-1 (SDF-1) were measured by enzyme-linked immunosorbent assay on stored plasma (EDTA) specimens as per the manufacturer's instructions (R&D systems).

### Statistical analyses

Results are expressed as mean±SD unless otherwise indicated. Baseline variables were analyzed with chi-square test for categorical variables, and *t* test for continuous variables. Correlations between EPC number and function, CAD severity, growth factors and appearance of OEC colonies (in days) were assessed using the Spearman rank test. The robustness of the relationship between CAD severity parameters and EPC counts and function when adjusted for age was assessed by partial correlation analysis. Separate subgroup analyses were also performed for the following potential confounders: gender, diabetes, smoking status, statin and angiotensin-converting enzyme inhibitor (ACEI) or angiotensin receptor blocker (ARB) use. These variables were chosen as they have been previously reported to influence EPC levels [5]. A multivariable analysis was not performed given the small number of patients and relatively large number of independent variables. Good interobserver reliabilities for stenosis severity, corrected TIMI frame count and TIMI myocardial perfusion grade were demonstrated, with weighted κ scores of 0.72, 0.63 and 0.71 respectively. A total of 30 patients was calculated to provide >80% power to detect correlations of >0.5. A two-sided p value <0.05 was considered to indicate statistical significance. Statistical analyses were performed using SPSS version 21 (IBM).

## Results

Thirty-three patients were recruited for the study, with a mean age of 62±10 (range 42–84) years. Twenty-seven (82%) were men. With regards to epicardial CAD characteristics, the mean stenosis severity was 68±29% (range 0-99%) and the mean FFR was 0.60±0.19 (range 0.23–0.89). There was a negative correlation between FFR and both LAD stenosis severity (r = -0.837, p<0.001) and Gensini score (r = -0.527, p = 0.001). With regards to the coronary microcirculation, the mean IMR was 22±13 (range 0–64.6), with 20 (61%) patients having IMR>20, a cut-off which has been proposed as being abnormal [23]. The rest of the baseline patient characteristics are summarized in Table 1.

**Table 1:** Baseline patient characteristics.

Patient variable	Mean±SD
**Risk factors**	
Age	62±10
Male*	27 (82)
Hypertension*	22 (67)
Diabetes*	9 (27)
Hypercholesterolemia*	26 (79)
Current smoker*	6 (18)
Family history of CAD*	12 (36)
**Past history**	
Previous myocardial infarction*	5 (15)
Previous stroke*	5 (15)
Previous angioplasty*	4 (12)
Renal dysfunction*	0 (0)
**Medications**	
Statin*	26 (79)
ACEI/ARB*	22 (67)
**Coronary physiology parameters**	
FFR	0.60±0.19
CFR	2.15±1.07
IMR	22±13
**Angiographic characteristics**	
Stenosis severity	68±29
Modified Gensini score	76±31
Corrected TIMI frame count	17.5±6.6
TIMI myocardial perfusion grade	2.7±0.6

Unless specified, results expressed as mean±SD.

*n (%).

### Relationship between epicardial coronary artery disease severity and endothelial progenitor cell number and function

**Figure 1:**
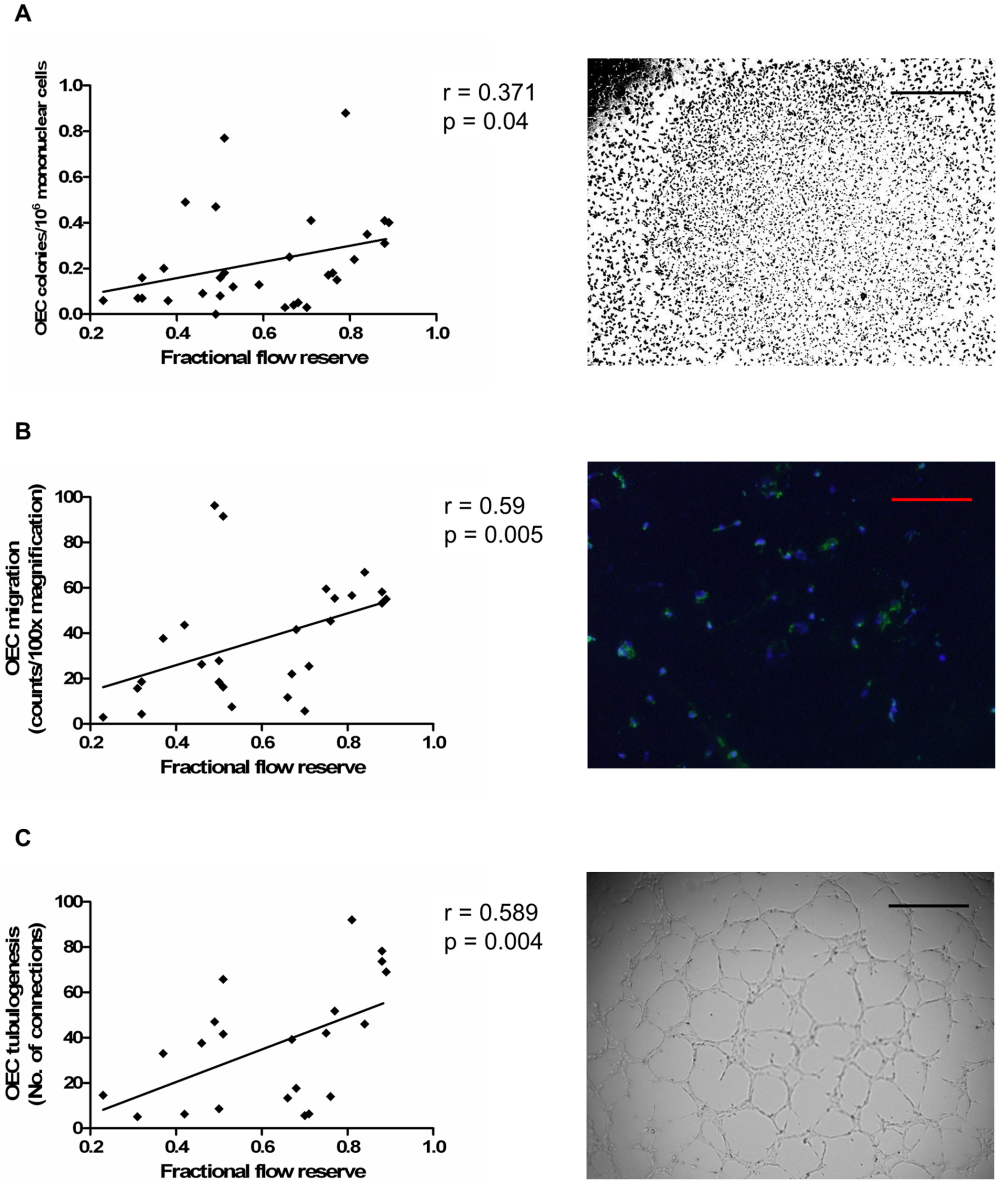
Higher circulating OEC levels and function are associated with less severe epicardial CAD, as assessed by FFR. (A) Association between OEC levels and FFR. (B) Association between OEC migration and FFR. (C) Association between OEC tubulogenesis and FFR. Correlations were assessed using the Spearman rank test. Images on the right panel show (A) OEC colony; scale bar, 100 µm, (B) OECs migrating onto transwell membrane, identified by co-staining with *Ulex europaeus* lectin (green) and DAPI (blue); scale bar, 50 µm, (C) network formation on Matrigel by OECs; scale bar, 100 µm.

**Figure 2:**
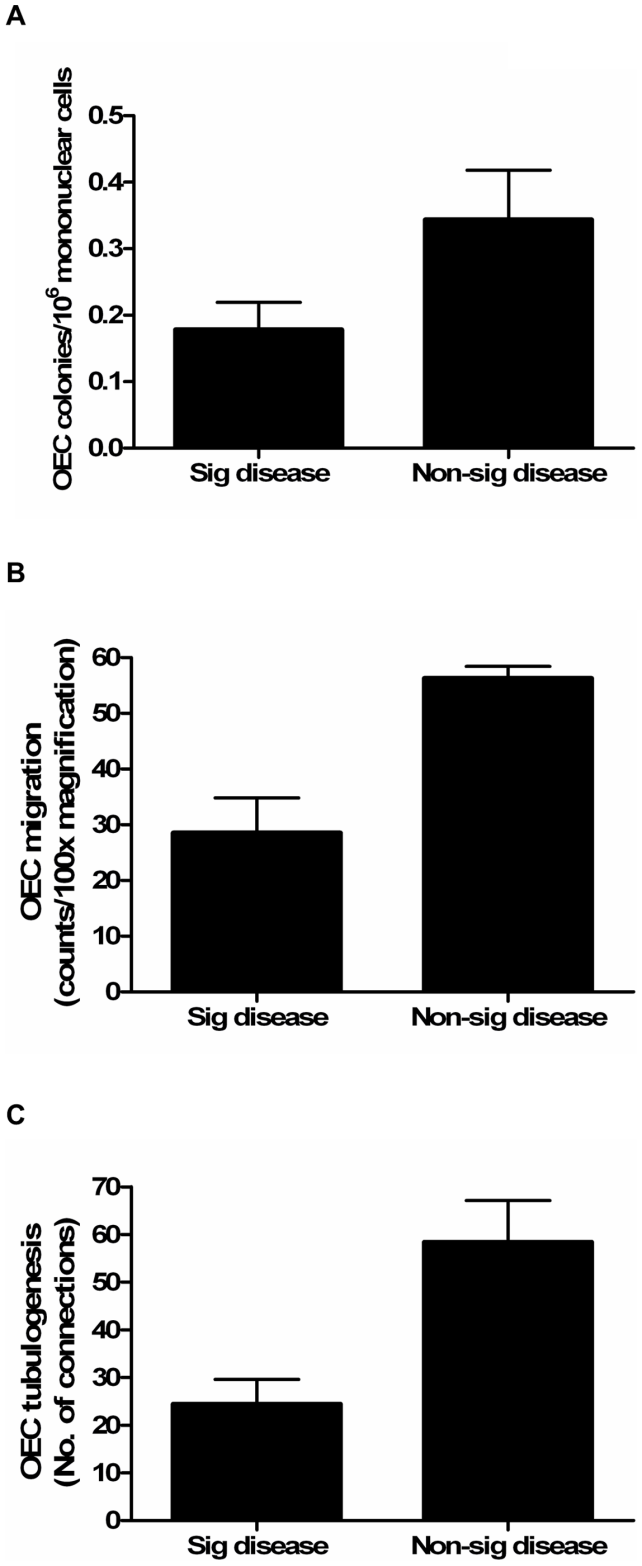
Circulating OEC levels and function according to significant (FFR<0.75) and non-significant (FFR≥0.75) LAD disease. Patients with significant LAD disease (FFR<0.75) had; (A) lower OEC levels (p<0.05), (B) impaired OEC migration (p<0.01), and (C) OEC tubulogenesis (p<0.01) compared with those with non-significant disease. The comparison between groups was assessed using the Student's *t* test. Data are expressed as mean±SEM. Sig, significant.

The relationship between EPC levels and epicardial CAD severity was assessed by physiology (FFR) and angiography (stenosis severity and modified Gensini score). We found that more severe epicardial CAD, as assessed by FFR, was associated with lower circulating culture-derived OEC levels (r = 0.371, p = 0.04, Figure 1) and impaired OEC function, as assessed by migration and tubulogenesis (r = 0.59, p = 0.005; r = 0.589, p = 0.004; respectively, Figure 1). Compared with patients with hemodynamically non-significant epicardial CAD (FFR≥0.75), those with significant disease (FFR<0.75) had lower OEC levels and impaired OEC function (p<0.05 for all, Figure 2). There was however no significant correlation between FFR and OEC proliferation (r = 0.012, p = 0.96).

**Figure 3:**
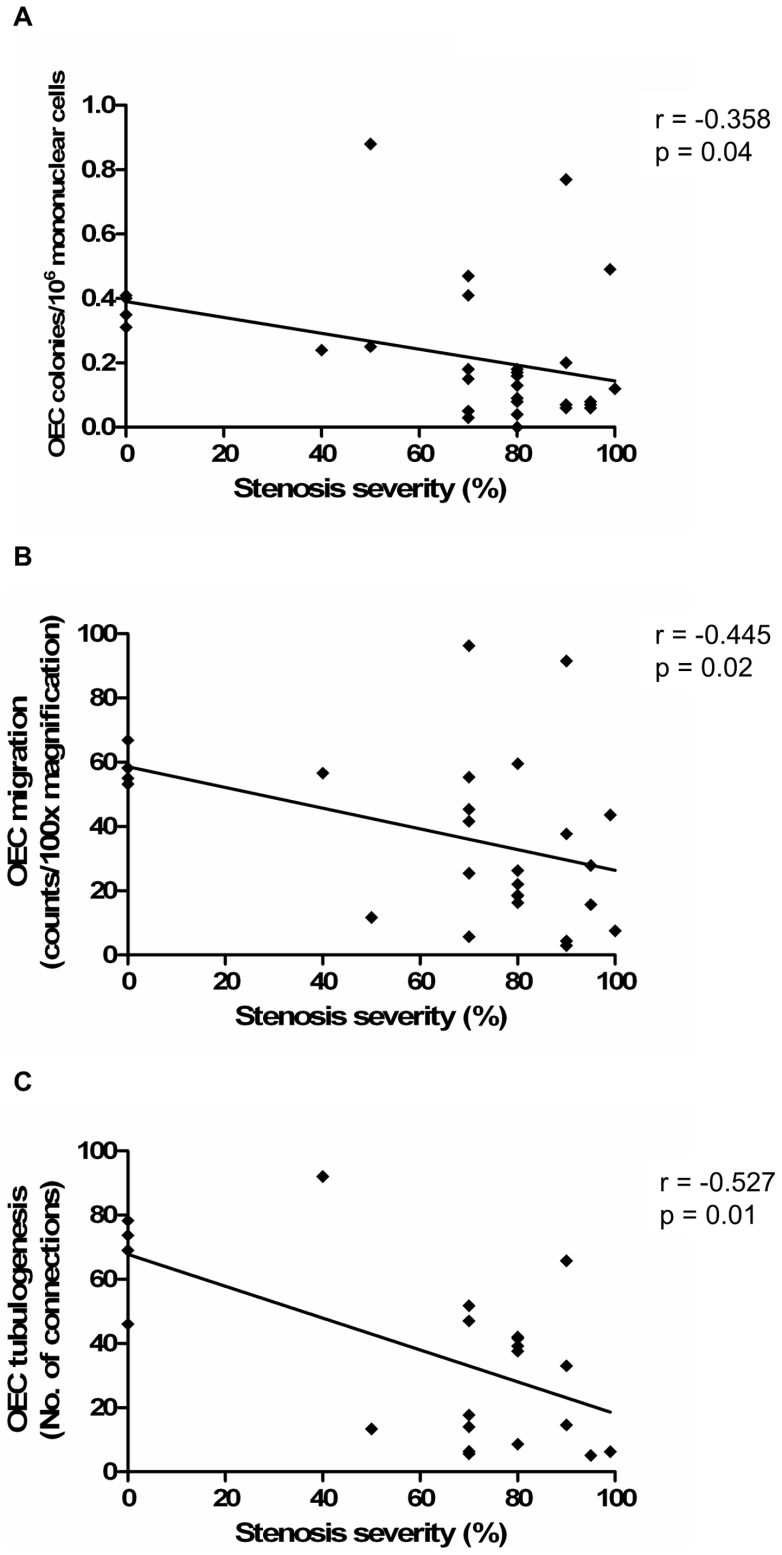
Higher circulating OEC levels and function are associated with less severe epicardial CAD, as assessed by angiography. (A) Association between angiographic LAD stenosis severity and OEC levels. (B) Association between LAD stenosis and OEC migration. (C) Association between LAD stenosis and OEC tubulogenesis. Correlations were assessed using the Spearman rank test.

Similar findings were also found with regards to LAD stenosis severity, whereby more severe LAD stenosis was associated with lower OEC levels (r = -0.358, p = 0.04, Figure 3) and impaired OEC function, as assessed by migration and tubulogenesis (r = -0.445, p = 0.02; r = -0.527, p = 0.01; respectively, Figure 3). Again, there was no significant relationship between LAD stenosis severity and OEC proliferation (r = 0.021, p = 0.93). The relationship with the modified Gensini score was only significant for OEC tubulogenesis (Table 2). There was no significant relationship between the number of days it took for OEC colonies to appear after plating, and OEC migration (r = -0.268, p = 0.20), tubulogenesis (r = 0.126, p = 0.59) and proliferation (r = -0.057, p = 0.81).

**Table 2:** Relationship between circulating EPC levels and function and epicardial CAD severity.

	FFR		Stenosis severity		Modified Gensini score	
	Spearman r	p value	Spearman r	p value	Spearman r	p value
**EPC levels**						
Early EPC	0.198	0.27	-0.126	0.48	-0.127	0.47
OEC	0.371	0.04	-0.358	0.04	-0.288	0.11
CD34^+^/KDR^+^	-0.169	0.39	0.073	0.71	0.299	0.12
CD34^+^/CD45^+^	0.179	0.35	-0.169	0.37	-0.142	0.46
CD34^+^/CD45^−^	-0.102	0.6	0.077	0.68	0.051	0.79
**EPC function**						
Early EPC migration	0.292	0.14	-0.286	0.14	-0.202	0.3
Early EPC tubulogenesis	0.347	0.15	-0.237	0.31	-0.205	0.39
OEC migration	0.59	0.005	-0.445	0.02	-0.072	0.73
OEC tubulogenesis	0.589	0.004	-0.527	0.01	-0.484	0.02
OEC proliferation	0.012	0.96	0.021	0.93	-0.116	0.61

**Figure 4:**
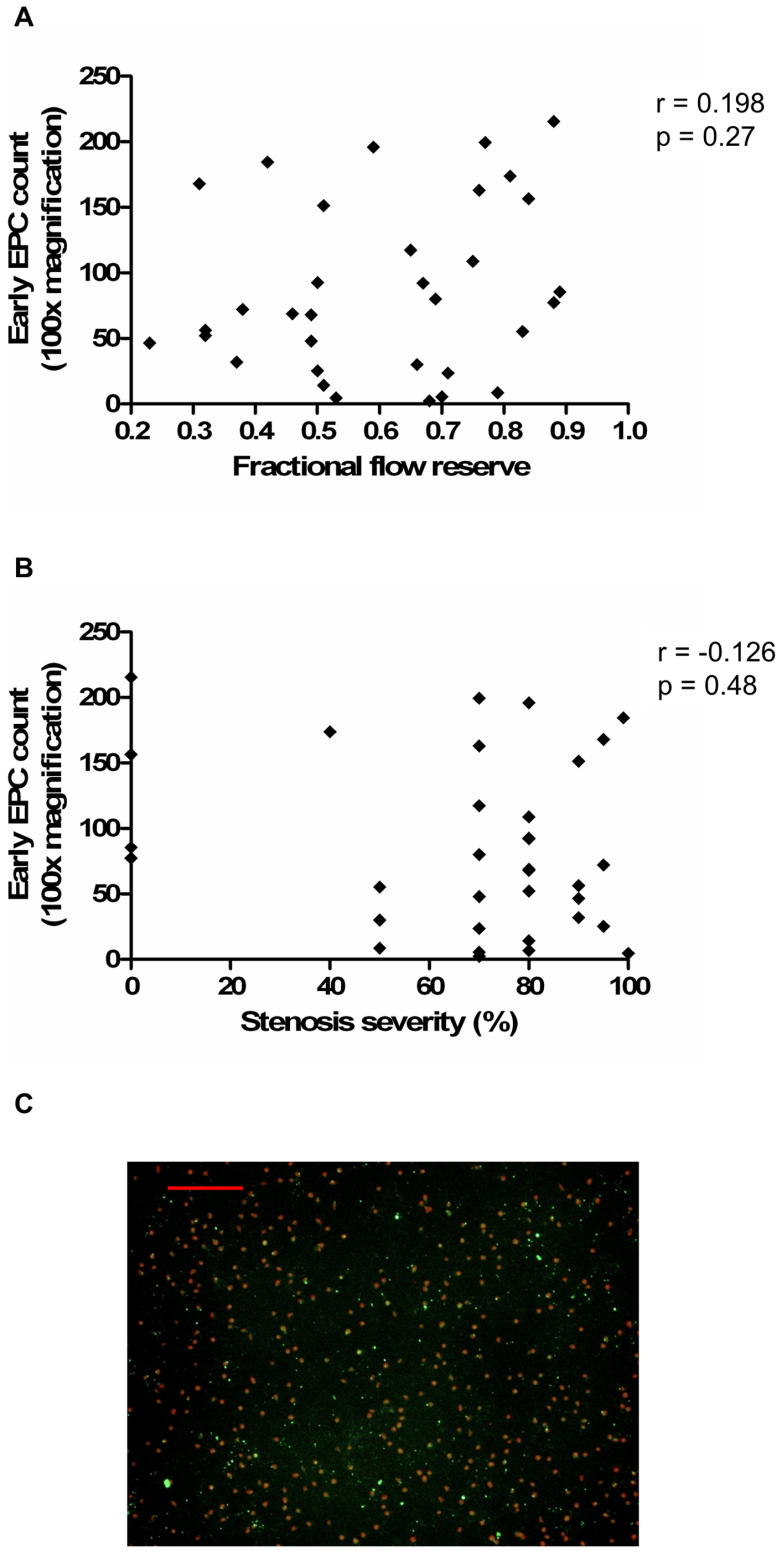
Circulating early EPC levels do not correlate with severity of epicardial CAD. (A) Association between early EPC levels and FFR. (B) Association between early EPC levels and stenosis severity. (C) Image example of early EPCs, identified by co-staining with Dil-Ac-LDL (red) and *Ulex europaeus* lectin (green); scale bar, 50 µm. Correlations were assessed using the Spearman rank test. Dil-Ac-LDL, 1,1′dioctadecyl-3,3,3′,3′-tetramethyl-indocarbocyanide-labeled acetylated low-density lipoprotein.

**Figure 5:**
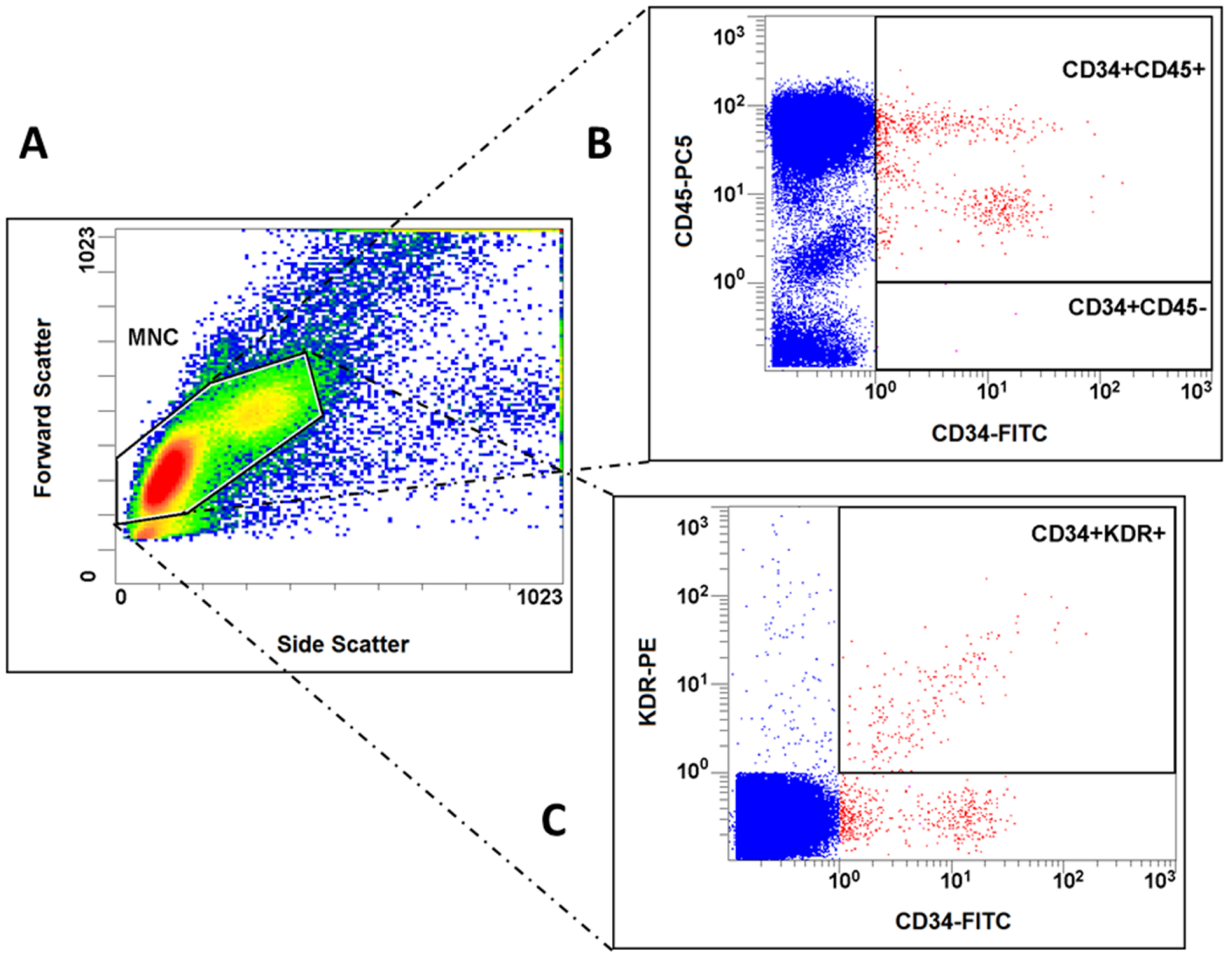
Flow cytometry-derived EPC definitions. (A) Cells isolated from whole blood by density gradient separation were gated by forward and side scatter to select mononuclear cells by excluding erythrocytes, granulocytes and cell debris. (B) The mononuclear-gated cells that stained positive for CD34-FITC were analyzed for the presence or absence of CD45-PC5. (C) Mononuclear cells were also analyzed for co-staining of CD34-FITC and KDR-PE. MNC, mononuclear cell; CD34-FITC, fluorescein isothiocyanate-conjugated CD34; CD45-PC5, phycoerythrin-Cy5-conjugated CD45; KDR-PE, phycoerythrin-conjugated KDR.

In contrast, there was no significant relationship between epicardial CAD severity, as assessed by FFR and stenosis severity, and culture-derived early EPC levels (Figure 4) and function (Table 2, with example of images shown in Figure S1). There was also no significant correlation between epicardial CAD severity and levels of EPCs identified on flow cytometry (Figure 5), including CD34^+^/KDR^+^ EPCs and enriched populations of early EPCs (CD34^+^/CD45^+^) and OECs (CD34^+^/CD45^−^) (Table 2).

### Relationship between coronary microvascular integrity and endothelial progenitor cell number and function

The state of the coronary microcirculation was assessed by physiology (IMR and CFR) and angiography (TIMI myocardial perfusion grade and corrected TIMI frame count). Consistent with previous studies, we found a strong correlation between CFR and corrected TIMI frame count (r = -0.58, p<0.001) [20].

**Figure 6:**
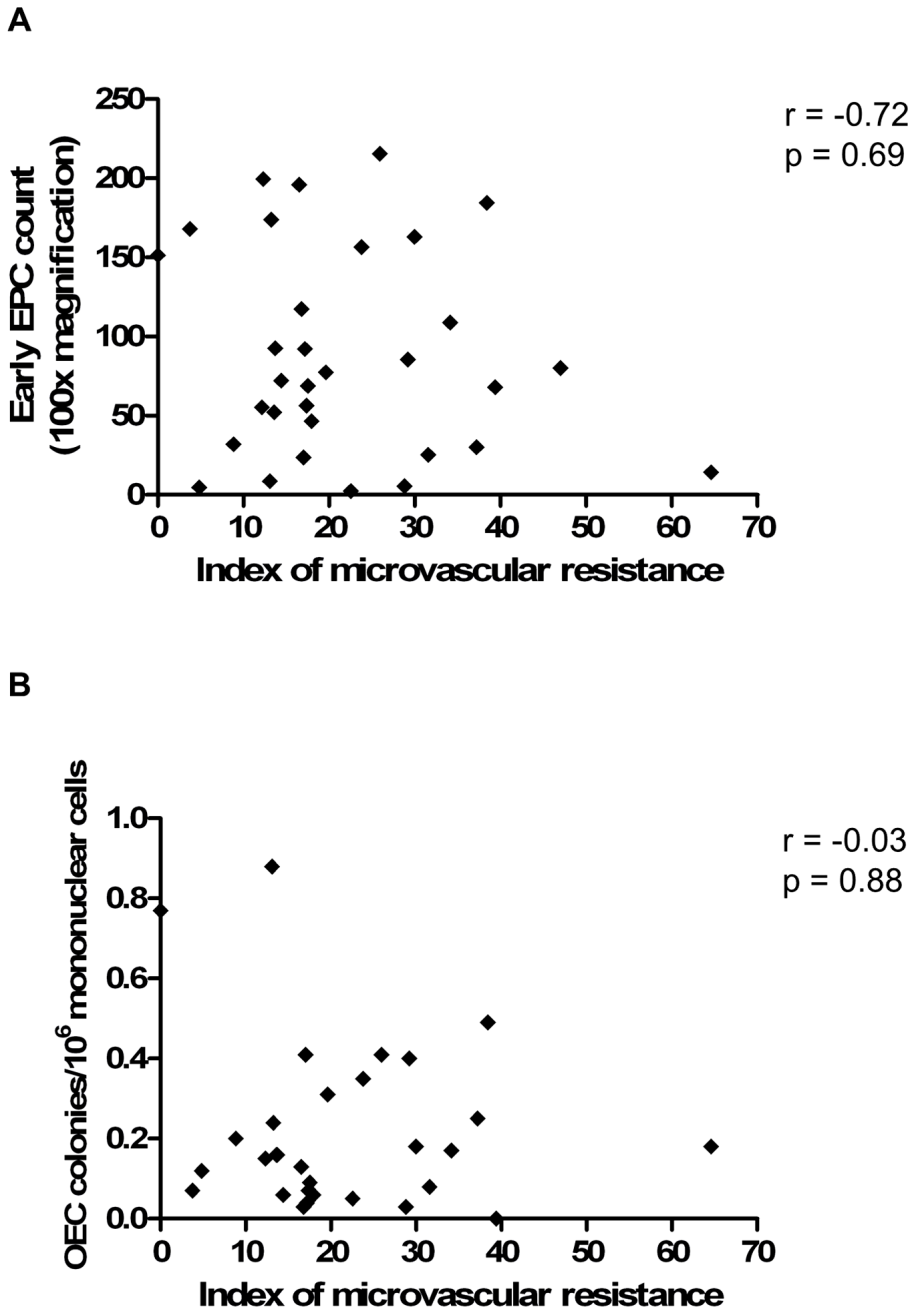
There is no correlation between circulating EPC levels and coronary microvascular integrity, as assessed by IMR. (A) Association between early EPC levels and IMR. (B) Association between OEC levels and IMR. Correlations were assessed using the Spearman rank test.

We did not find a significant relationship between microvascular disease severity, as assessed by IMR (a specific measure of microvascular integrity), and culture-derived early EPC and OEC levels (Figure 6) and function (Table 3). There was also no significant relationship between IMR and levels of flow cytometry-defined EPCs (Table 3).

**Table 3:** Relationship between circulating EPC levels and function and coronary microvascular integrity.

	IMR		CFR		TIMI myocardial perfusion grade		CTFC	
	Spearman r	p value	Spearman r	p value	Spearman r	p value	Spearman r	p value
**EPC levels**								
Early EPC	-0.72	0.69	0.257	0.16	0.15	0.49	-0.15	0.39
OEC	-0.03	0.88	0.332	0.07	0.32	0.08	0.15	0.41
CD34^+^/KDR^+^	0.051	0.8	-0.226	0.25	0.064	0.75	-0.027	0.89
CD34^+^/CD45^+^	0.122	0.53	0.151	0.44	-0.25	0.19	-0.327	0.08
CD34^+^/CD45^−^	-0.002	0.99	-0.158	0.41	-0.048	0.8	0.115	0.55
**EPC function**								
Early EPC migration	0.051	0.8	0.246	0.22	0.052	0.8	-0.105	0.6
Early EPC tubulogenesis	-0.125	0.61	0.3	0.21	-0.031	0.9	0.042	0.87
OEC migration	0.042	0.84	0.499	0.01	0.38	0.06	0.023	0.91
OEC tubulogenesis	-0.084	0.72	0.573	0.007	0.19	0.41	-0.23	0.3
OEC proliferation	0.216	0.35	0.087	0.71	-0.191	0.41	0.092	0.69

CTFC, corrected TIMI frame count.

**Figure 7:**
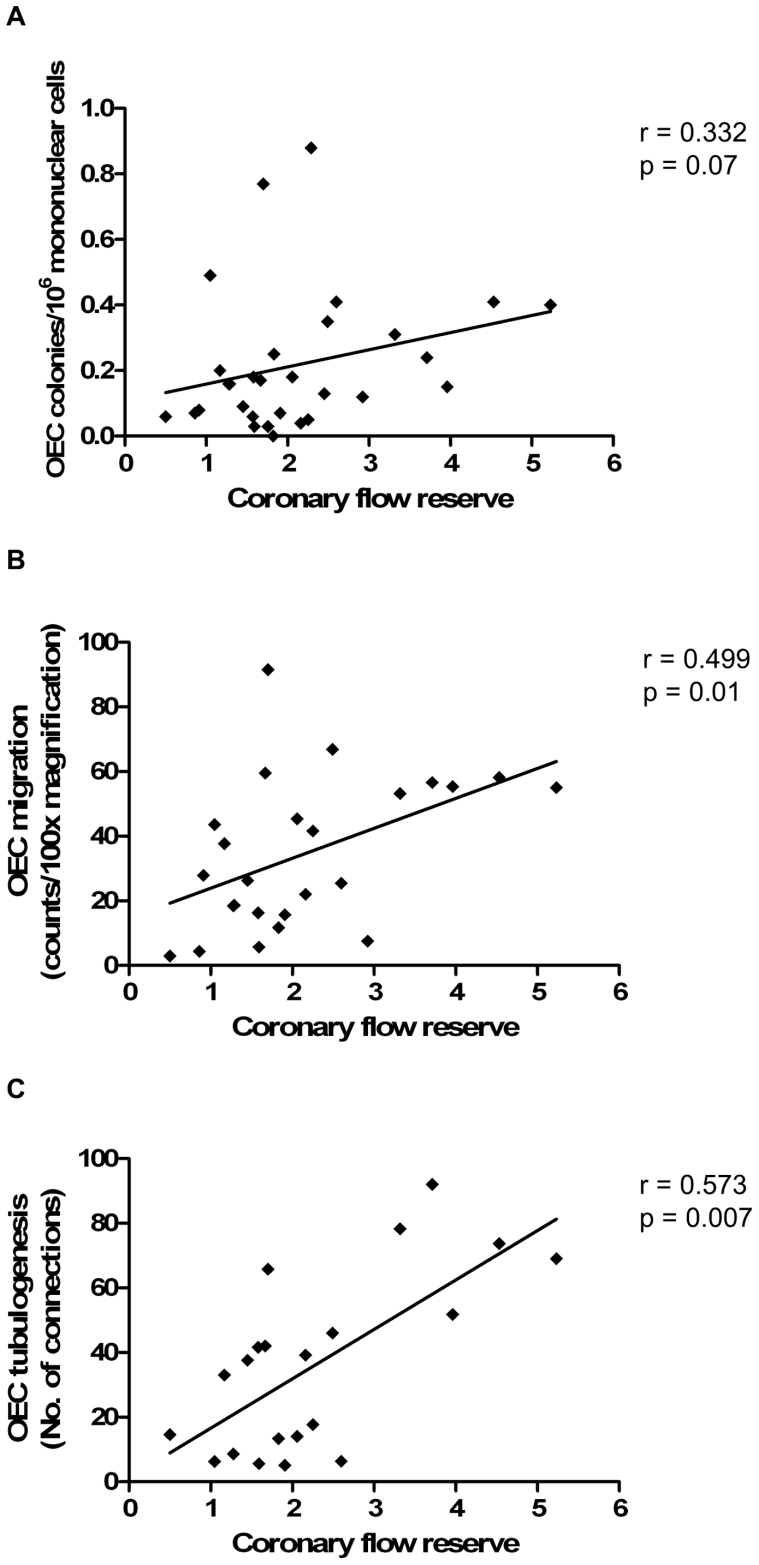
The relationship between circulating OEC level and function and CFR. (A) Association between OEC levels and CFR. (B) Association between OEC migration and CFR. (C) Association between OEC tubulogenesis and CFR. Correlations were assessed using the Spearman rank test.

When the coronary microvasculature was assessed by CFR, there was a trend towards a positive correlation between CFR and culture-derived OEC levels, although this was not statistically significant (r = 0.332, p = 0.07, Figure 7). There was also a positive correlation between CFR and OEC function, as assessed by migration and tubulogenesis (r = 0.499, p = 0.01; r = 0.573, p = 0.007; respectively, Figure 7). However, when adjusted for FFR using partial correlation analyses, these associations were no longer significant or trending, suggesting that the relationships with OEC levels and function are likely driven by the epicardial component of the CFR measure. There was no significant correlation between CFR and OEC proliferation (r = 0.087, p = 0.71). In addition, no such relationship was seen for early EPC function.

No significant correlation was seen with regards to the angiographic assessment of coronary microvascular integrity (TIMI myocardial perfusion grade and corrected TIMI frame count) and EPC number and function (Table 3).

### Relationship between growth factors and EPC number and function

As growth factors have been implicated in regulation of EPC mobilization and function [24], we assessed the relationship between concentrations of growth factors and EPC, and found that there was no significant correlation between plasma VEGF_165_ and SDF-1 concentration and EPC number and function (Table 4). There was also no significant correlation between growth factor concentrations and FFR, IMR or CFR.

**Table 4:** Relationship between growth factors and EPC number and function.

	VEGF_165_		SDF-1	
	Spearman r	p value	Spearman r	p value
**EPC levels**				
Early EPC	-0.023	0.9	0.042	0.82
OEC	-0.167	0.38	-0.182	0.34
CD34^+^/KDR^+^	0.318	0.11	-0.112	0.58
CD34^+^/CD45^+^	-0.132	0.5	0.013	0.95
CD34^+^/CD45^−^	-0.055	0.78	0.059	0.77
**EPC function**				
Early EPC migration	-0.342	0.09	-0.233	0.25
Early EPC tubulogenesis	0.012	0.96	0.011	0.97
OEC migration	-0.157	0.46	0.062	0.77
OEC tubulogenesis	-0.094	0.69	0.031	0.89
OEC proliferation	0.161	0.5	0.006	0.98

### Adjusted and subgroup analyses

When adjusted for age, the relationship between circulating OEC levels and FFR and stenosis severity was no longer statistically significant, although a similar relationship remained (r = 0.289, p = 0.12; r = -0.34, p = 0.06; respectively). With regards to EPC function, the relationship between FFR and OEC function persisted after adjusting for age (r = 0.42, p = 0.04; r = 0.49, p = 0.02; for migration and tubulogenesis respectively). The relationship between stenosis severity and OEC tubulogenesis also persisted after adjusting for age (r = -0.59, p = 0.005); however, the relationship between stenosis severity and OEC migration was no longer significant when adjusted for age, although again the relationship remained similar (r = -0.38, p = 0.06). Likewise, the positive correlation between CFR and OEC migration and tubulogenesis persisted after adjusting for age (r = 0.44, p = 0.03; r = 0.64, p = 0.002; respectively). There was also no significant correlation between OEC proliferation and either migration (r = -0.116, p = 0.62) or tubulogenesis (r = -0.015, p = 0.95), suggesting that the OEC migration and tubulogenesis data were not influenced by proliferation during the course of the assay.

In subgroup analyses separately by gender, diabetes, smoking status, statin and ACEI/ARB use, the correlation coefficients between OEC levels/function and FFR/stenosis severity remained essentially similar, although statistical significance was not reached in the smaller subgroups.

## Discussion

In a systematic assessment of the relationship between the number and function of distinct EPC populations and angiographic and physiologic extent of CAD severity, our key findings are that patients with more severe epicardial CAD had impaired migration and tubulogenesis function and lower levels of culture-derived OECs. In contrast, no such relationship was found between epicardial CAD severity and culture-derived early EPC number and function, as well as number of CD34^+^/KDR^+^ EPCs and enriched populations of early EPCs (CD34^+^/CD45^+^) and OECs (CD34^+^/CD45^−^). In addition, there was no correlation between EPC number and function and the state of the coronary microcirculation. Interestingly, the relationship between epicardial CAD severity and OEC function, but not number, persisted after adjusting for age, raising the hypothesis that progenitor cell function plays a more important role in protection from epicardial vessel disease than the actual number of OECs. These findings are consistent with an earlier study reporting that patients with chronic ischemic cardiomyopathy had impaired function, but not levels, of bone-marrow derived progenitor cells, compared with normal healthy controls [25].

The role of EPCs in atheroprotection remains poorly understood and controversial. Experiments using animal models of atherosclerosis have yielded conflicting results. In one study, long-term treatment with bone marrow-derived EPCs from young non-atherosclerotic Apolipoprotein E (*ApoE*) knockout mice prevented atherosclerosis progression in *ApoE* knockout recipients despite persistent hypercholesterolemia [2]. However, other studies reported contrasting results, and found that transplantation of EPCs increased atherosclerotic plaque progression and lesion size in *ApoE* knockout mice [26], [27].

Likewise, previous studies exploring the relationship between EPC levels and epicardial CAD severity in humans have also yielded conflicting results. Consistent with our findings, Kunz *et al* reported an inverse correlation between EPC colony forming unit levels and CAD severity [4]. In contrast, Güven *et al* reported that patients with angiographically more severe CAD had higher levels of OEC and early EPC compared with those with less severe CAD [3]. Another study also reported higher levels of CD34^+^/KDR^+^ EPCs and CD34^+^/CD133^+^ EPCs in patients with severe angiographic coronary stenosis compared with those with normal coronary arteries [28]. More recently, Padfield *et al* reported that increased CD34^+^/CD45^−^ EPCs were associated with more severe CAD (assessed using the Gensini score), and interestingly, also better clinical outcomes [29]. However, studies assessing the non-coronary circulation have reported a similar association with our study and findings by Kunz *et al*, whereby lower EPC levels were associated with more severe peripheral vascular disease and greater carotid intima-media thickness [24], [30], [31].

A recent study assessing the biological function of OECs also reported results consistent with our study, whereby patients with CAD had impaired OEC endothelial phenotype expression and tubulogenesis when compared with healthy controls [32]. Studies exploring EPC counts and function in patients with pulmonary arterial hypertension (PAH) have also been undertaken, with one study reporting increased CD34^+^/KDR^+^/CD133^+^ EPC levels in patients with PAH, and reduced OEC tubulogenesis and increased proliferation in PAH patients with the bone morphogenetic protein receptor type II mutation [33], and another study reporting increased OEC levels in PAH patients treatment with treprostinil (a parenteral prostanoid) [34]. More recently, patients with exacerbation of idiopathic pulmonary fibrosis were reported to be associated with increased OEC proliferation [35]. In our study, we did not find a relationship between OEC proliferation and CAD severity, although this disparate result may be related to distinct pathogenic mechanisms underlying CAD and pulmonary disease.

There are a number of potential reasons for the conflicting results. Firstly, the previous studies relied solely on angiography to assess CAD severity, a technique which may have limited correlation with the physiological significance of disease severity [6]. Although EPC number and functionality are assumed to reflect the endogenous vascular protective capacity, an alternative explanation for these conflicting results may be that severe CAD results in ischemia and a pro-inflammatory state, thus leading to EPC mobilization. This explanation is supported by studies showing increased EPC number and clonogenic expansion capacity *in vitro* in patients suffering from acute coronary syndrome [36], [37], [38], [39]. Consistent with the earlier studies, Padfield *et al* recently reported that levels of CD34^+^/CD45^−^ EPCs, CD14^+^/KDR^+^/Tie-2^+^ EPCs and colony forming unit endothelial cells (CFU-ECs) were higher in patients with acute coronary syndrome compared with those with stable angina or normal coronary arteries [29]. The heterogeneous nature of the subjects in the study by Padfield *et al* [29], which included both patients with stable and unstable CAD, may also explain the disparate results between their study and our study, whereby we did not observe a relationship between CD34^+^/CD45^−^ EPCs and Gensini score.

Secondly, the EPC definitions between studies were not uniform. Key biological differences exist between EPC populations. OECs, in contrast with early EPCs, have high proliferative potential [8], [10]. Yoder *et al* reported that human OECs form perfused vessels *in vivo* when transplanted into immunodeficient mice [15]. In contrast, early EPC colony forming units were derived from the hematopoietic system and failed to form perfused vessels [15]. In addition, another study reported that early EPCs and OECs exhibit different properties *in vitro*, with OECs directly participating in tubulogenesis and early EPCs augmenting tubulogenesis in a paracrine fashion instead [14]. These results support our findings, where we found that higher OEC levels were associated with less severe CAD. Lastly, other potential reasons for the conflicting results include significant variability between studies in the time point at which early EPC counts were performed, ranging from 4 to 12 days post culture, and the fact that EPC levels may exhibit diurnal variation [24], so that the differing relationships reported may simply reflect different blood collection times.

Our study also explored the relationship between EPCs and the physiologic status of the coronary microcirculation, a critical determinant of cardiovascular outcomes [7]. Previous studies on patients with cardiac syndrome X (*i.e*. the presence of angina with abnormal ischemia testing and normal coronary angiogram, which is thought to be due to coronary microvascular disease) have yielded conflicting results with regards to EPC levels, with one study reporting higher EPC levels, and another reporting decreased levels and adhesive function of circulating EPCs, when compared to normal controls [40], [41]. Another more recent study involving patients post myocardial infarction reported that the presence of OEC was associated with reduced microvascular obstruction and infarct size, as assessed by cardiac magnetic resonance imaging (MRI), suggesting that OEC may be a marker of microvascular integrity [42]. Additionally, EPCs have been shown to correlate with the extent of coronary collateralization by qualitative angiography [28]. Apart from the non-uniform EPC definitions, a possible reason for the disparate results between our present study (where we found no relationship between EPCs and IMR) and the previous studies was that the state of the coronary microcirculation was not physiologically assessed in the previous studies. In the studies involving patients with cardiac syndrome X, the diagnosis of microcirculatory dysfunction was presumed on the basis of exclusion. Assessment of coronary microvascular dysfunction by cardiac MRI may also be compounded by the presence of concomitant epicardial coronary disease [43]. Although we did find an association between OEC function and CFR, this is likely driven by the epicardial rather than the microvascular component of this measurement as this association was no longer significant when adjusted for FFR. Consistent with our findings, Fadini *et al* also did not find any relationship between EPC levels and the microvascular complications of diabetes (such as retinopathy and nephropathy) [30]. This apparently disparate result between the coronary epicardial and microcirculation may be due to the different pathogenic mechanisms leading to the development of epicardial and microvascular disease. Given the small number of patients in this study, it is possible that a significant association between OEC levels and CFR could have been missed. Likewise, the relationship between OEC levels and function and the microvasculature remains to be clarified by a larger study.

### Study strengths and limitations

There are several limitations of our study. This is a relatively small study comprising only 33 patients, hence it is possible that additional correlations (*e.g.* between EPCs and coronary microvascular disease) may have been missed. However, our study only assessed patients with a spectrum of LAD disease without significant disease elsewhere, thereby minimizing any confounding factors which may have arisen from assessment of other coronary artery territories. In addition, the strength of the observed correlations was relatively modest and thus best regarded as hypothesis-generating.

We did not assess EPCs for the cell surface marker CD133 because, while it has been suggested that CD34^+^/KDR^+^/CD133^+^ cells may represent a population of cells with progenitor properties, others have found that purified CD34^+^/KDR^+^/CD133^+^ cells are highly enriched in hematopoietic progenitor activity and do not give rise to any OEC colonies *in vitro* [9], [44]. Instead, we assessed for the cell surface marker CD45 as it has been reported that CD133 and CD45 often coexist, and that CD34^+^/CD45^+^ and CD34^+^/CD45^−^ cells may represent enriched populations of early EPCs and OECs respectively [9]. Functional assays were not performed on EPCs thus identified by flow cytometry, as cell counts were too low to permit culture and expansion of these cells for functional assessment. Another putative population of EPCs, CFU-ECs isolated by the endocult method, have been reported to be inversely correlated with cardiovascular risk factors and mortality [45], [46]. However, it has been shown that these CFU-ECs derive from the hematopoietic system and fail to form perfused vessels *in vivo* when transplanted into immunodeficient mice [15]. We did not explore the relationship between CAD severity and this population of EPCs.

Our study does not provide a mechanism for the observed association between OEC levels and function and epicardial CAD severity. In contrast with experimental studies reporting the role of growth factors (such as VEGF or SDF-1) in EPC differentiation, mobilization and homing [24], we did not find a correlation between growth factor concentrations, EPC number and function and CAD severity. There were also significant missing data due to insufficient cell numbers or contamination, especially with regards to OEC tubulogenesis assay (n = 11). However, there were no statistically significant differences in baseline characteristics between patients with and without missing OEC tubulogenesis data, suggesting that the inadequate cell numbers or contamination were both random phenomena (Table S1). We also acknowledge that we did not assess OEC clonogenic ability by cell limit dilution, which could have yielded different results to the nucleotide incorporation proliferation assay used in this study. Finally, the association reported here is not necessarily causal, and future studies on OEC transplantation *in vivo* may better elucidate the role of OECs in the pathogenesis of atherosclerosis.

The strengths of our study include its comprehensive assessment of both number and function of different EPC populations, the detailed angiographic and hemodynamic assessment of both coronary epicardial and microcirculation, as well as the exclusive interrogation of the LAD.

## Conclusion

We report that putative EPC populations exhibit differing relationships with the hemodynamic and angiographic extent of CAD in humans. Specifically, the number and function of OECs are inversely correlated with epicardial CAD severity. No such relationship exists for early EPCs. There was also no relationship between different EPC subtypes and coronary microvascular severity. Our data raises the hypothesis for the role of these cells in atheroprotection, with potential for optimizing cell therapy in the treatment of epicardial coronary disease.

## Supporting Information

Figure S1 **Examples of images obtained for early EPC migration and co-cultured tubulogenesis.** (A) Early EPCs migrating onto transwell membrane, identified by co-staining with *Ulex europaeus* lectin (green) and DAPI (blue); scale bar, 50 µm. (B) Early EPCs associating with HUVEC network formation on Matrigel; scale bar, 100 µm.
(DOCX)

Table S1 **Baseline characteristics between patients with and without missing data for OEC tubulogenesis.**
(DOCX)
