# Supplementary material for: The Relationship between Endothelial Progenitor Cell Populations and Epicardial and Microvascular Coronary Disease—A Cellular, Angiographic and Physiologic Study
Source: PLoS One. 2014 Apr 15;9(4):e93980. doi: 10.1371/journal.pone.0093980 (PMC3988011; doi:10.1371/journal.pone.0093980)
Supplement: Figure S1 — Examples of images obtained for early EPC migration and co-cultured tubulogenesis. (A) Early EPCs migrating onto transwell membrane, identified by co-staining with Ulex europaeus lectin (green) and DAPI (blue); scale bar, 50 µm. (B) Early EPCs associating with HUVEC network formation on Matrigel; scale bar, 100 µm. (DOCX) [file pone.0093980.s001.docx]

**Figure S1. Examples of images obtained for early EPC migration and co-cultured tubulogenesis.**

(A) Early EPCs migrating onto transwell membrane, identified by co-staining with *Ulex europaeus* lectin (green) and DAPI (blue); scale bar, 50 µm. (B) Early EPCs associating with HUVEC network formation on Matrigel; scale bar, 100 µm.


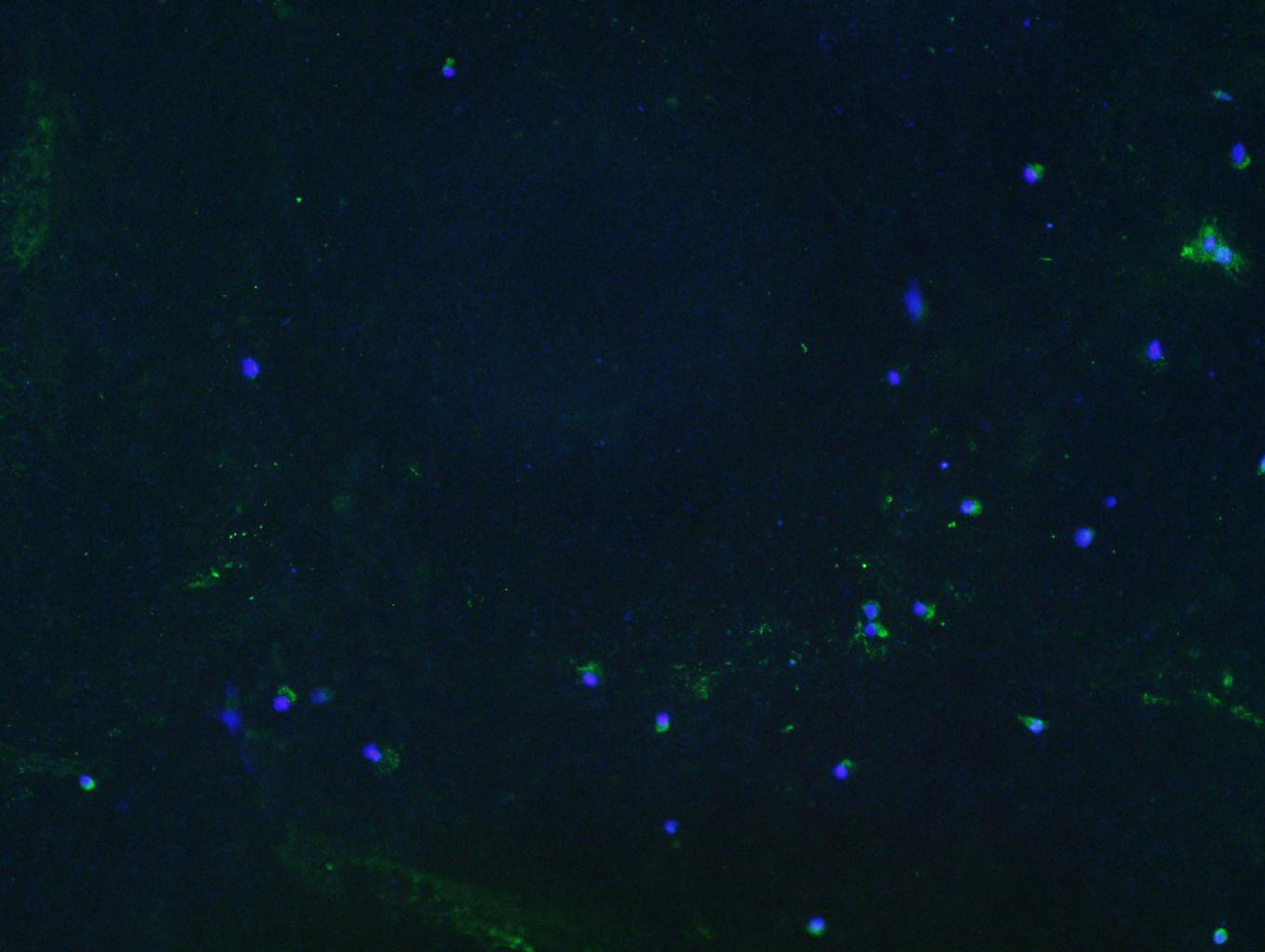

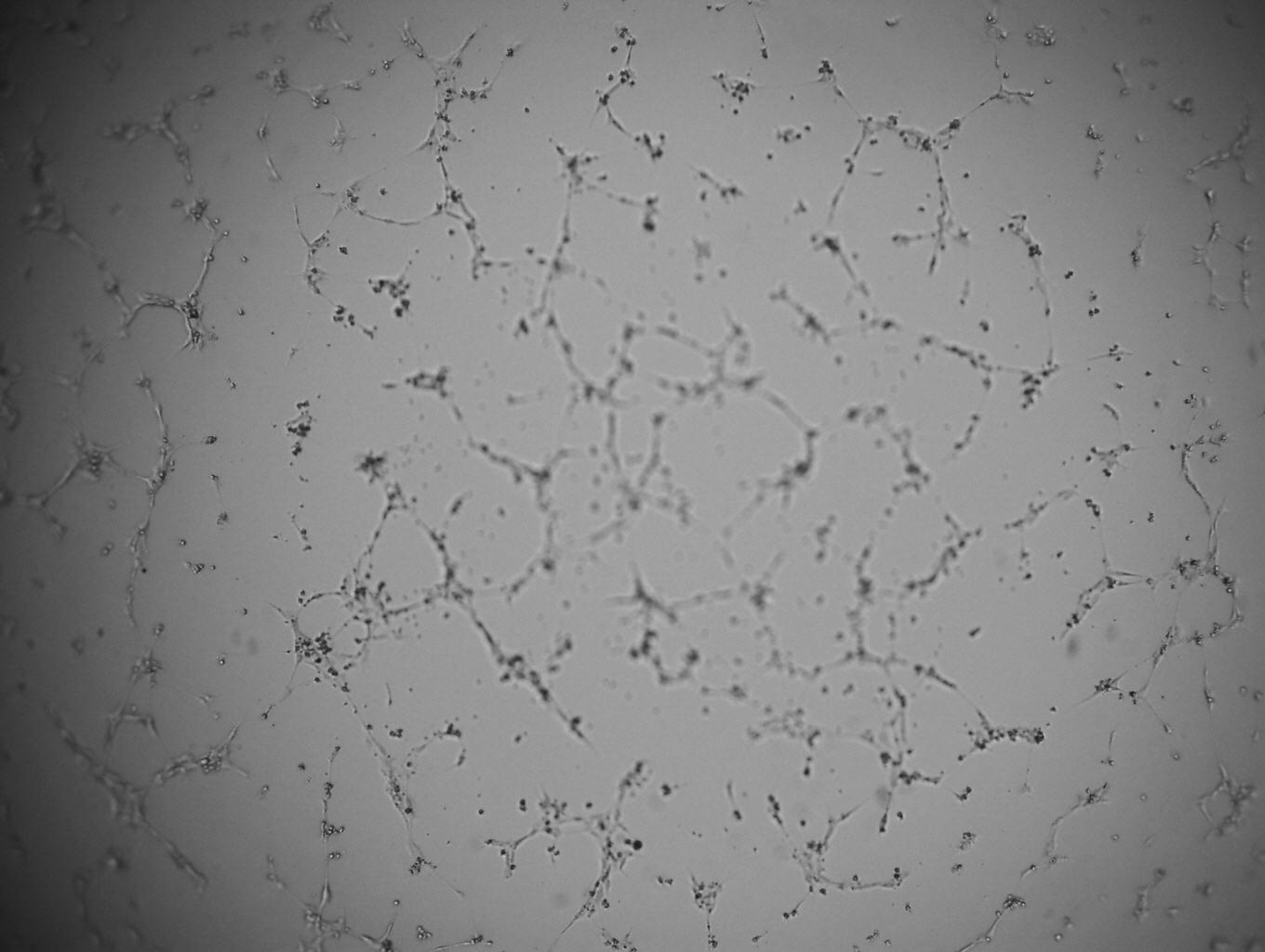


**A**

**B**
