# Supplementary material for: The Relationship between Endothelial Progenitor Cell Populations and Epicardial and Microvascular Coronary Disease—A Cellular, Angiographic and Physiologic Study
Source: PLoS One. 2014 Apr 15;9(4):e93980. doi: 10.1371/journal.pone.0093980 (PMC3988011; doi:10.1371/journal.pone.0093980)
Supplement: Table S1 — Baseline characteristics between patients with and without missing data for OEC tubulogenesis. (DOCX) [file pone.0093980.s002.docx]

**Table S1. Baseline characteristics between patients with and without missing data for OEC tubulogenesis**

| **Patient variable** | **Missing data**  **n = 11** | **No missing data**  **n = 22** | **p value** |
| --- | --- | --- | --- |
| **Risk factors** |  |  |  |
| Age | 63±9 | 61±11 | 0.74 |
| Male^*^ | 7 (64) | 19 (86) | 0.15 |
| Hypertension^*^ | 10 (91) | 12 (55) | 0.054 |
| Diabetes^*^ | 3 (27) | 6 (27) | 1.0 |
| Hypercholesterolemia^*^ | 9 (82) | 17 (77) | 1.0 |
| Current smoker^*^ | 2 (18) | 4 (18) | 1.0 |
| Family history of CAD^*^ | 2 (18) | 10 (45) | 0.25 |
| **Past history** |  |  |  |
| Previous myocardial infarction^*^ | 3 (27) | 2 (9) | 0.30 |
| Previous stroke^*^ | 2 (18) | 3 (14) | 1.0 |
| Previous PCI^*^ | 1 (9) | 3 (14) | 1.0 |
| **Medications** |  |  |  |
| Statin^*^ | 9 (82) | 17 (77) | 1.0 |
| ACEI/ARB^*^ | 10 (91) | 12 (55) | 0.054 |
| **Coronary physiology parameters** |  |  |  |
| FFR | 0.56±0.18 | 0.63±0.20 | 0.32 |
| CFR | 1.88±0.71 | 2.29±1.21 | 0.32 |
| IMR | 21±13 | 23±14 | 0.69 |
| **Angiographic characteristics** |  |  |  |
| Stenosis severity | 78±17 | 62±33 | 0.15 |
| Modified Gensini score | 85±29 | 71±31 | 0.24 |
| Corrected TIMI frame count | 17±6 | 18±7 | 0.55 |
| TIMI myocardial perfusion grade | 2.5±0.9 | 2.8±0.4 | 0.12 |

Unless specified, results expressed as mean±SD.

^*^n (%)

P values are from chi-square test for categorical variables, *t* test for continuous variables.
